# Scalable Fabrication of Highly Ordered Au/Ag Composite Nanoshell Array for Ultrasensitive SERS Detection of Trace Pesticides

**DOI:** 10.3390/nano16171109

**Published:** 2026-09-03

**Authors:** Jiabin Chen, Ye Dong, Shuyu Jiang, Tao Zhang, Zheng Liu, Kaiwen Hu, Zhiming Chen

**Affiliations:** School of Physical Science and Technology, Tiangong University, Tianjin 300387, China

**Keywords:** surface-enhanced Raman scattering sensing, Au/Ag nanoshell array, plasmonic coupling, self-assembly

## Abstract

Surface-enhanced Raman scattering is highly promising for trace molecular detection but requires substrates with both ultrahigh sensitivity and macroscopic uniformity. Current bimetallic nanostructures struggle to achieve large-area structural reproducibility and high-density “hot spots” due to the lack of precise spatial control over nanoparticle arrangement and the random distribution of nanogaps, which severely limits their quantitative reliability in realistic applications. Here, we developed a simple and scalable method to fabricate a highly ordered Au/Ag composite nanoshell array via combining the gas–liquid interface self-assembly and ion sputtering. This prepared substrate is featured by abundant nanoscale openings, crevices, and interfacial junctions. The Au/Ag composite nanoshell array achieves an ultra-low limit of 10^−11^ M for the standard probe Rhodamine 6G. The highly ordered array also ensures advanced large-area signal reproducibility with a relative standard deviation of 6.1%. This work successfully balances the large-area uniformity and high sensitivity in SERS detection. It also provides a robust and reliable plasmonic platform for practical food safety analysis and environmental monitoring.

## 1. Introduction

Surface-enhanced Raman scattering (SERS) represents a formidable analytical technique for trace molecular detection, distinguished by its ultrahigh sensitivity, rapid response, and capacity for unique molecular fingerprinting. Because SERS performance is fundamentally governed by localized surface plasmon resonance (LSPR) confined within nanoscale “hot spots” on noble metal substrates, this technique holds immense promise for food safety and environmental monitoring [1,2,3,4,5,6,7,8,9,10,11,12,13,14]. A prominent application lies in the rapid on-site screening of carbendazim [15,16,17], a widely employed broad-spectrum fungicide associated with severe ecological and health hazards [18,19,20,21]. Conventional analytical methods, such as liquid chromatography-tandem mass spectrometry, often necessitate cumbersome and time-consuming sample pretreatment protocols [22]. Consequently, the development of rapid and reliable SERS-based platforms for pesticide detection is of urgent practical significance.

Despite its vast potential, the translation of SERS into practical sensing applications is significantly impeded by bottlenecks in substrate design and scalable fabrication. Single-metal substrates inherently suffer from a critical performance trade-off: while silver (Ag) nanostructures afford massive electromagnetic enhancement, they oxidize rapidly under ambient conditions [23,24,25]; the gold (Au) ones provide superior chemical stability but exhibit inferior plasmonic activity [26,27,28,29,30,31]. To synergize these advantageous properties, bimetallic Au@Ag nanostructures have been extensively investigated [32,33,34,35,36]. Traditionally, such bimetallic materials are synthesized via colloidal methods and deposited by drop-casting. However, the stochastic aggregation intrinsic to this approach frequently induces the coffee-ring effect, culminating in severe spatial signal fluctuations. To achieve reliable quantitative analysis, researchers have increasingly focused on highly ordered plasmonic arrays, which afford advanced macroscopic signal uniformity. Nevertheless, a critical fabrication challenge persists. Although top-down lithographic techniques offer structural precision, their high cost and low throughput preclude scalable production. Conversely, conventional bottom-up self-assembly predominantly yields smooth periodic architectures that lack the abundant nanoscale crevices necessary for optimal field confinement. Owing to this lack of precise spatial control over nanogap distribution, current bimetallic platforms struggle to concurrently realize large-area structural reproducibility and high-density “hot spots” thereby severely limiting their quantitative reliability in realistic applications.

Here, we propose a facile and highly scalable methodology for fabricating a highly ordered Au/Ag composite nanoshell array by integrating gas–liquid interface self-assembly with sequential ion sputtering [37,38,39,40,41,42]. The self-assembled monolayer of polystyrene (PS) microspheres served as a structural template; followed by sequential sputtering deposition of Au and Ag. This tailored architecture not only yields a close-packed arrangement of nanoshells but also spontaneously generates abundant nanoscale openings, crevices, and interfacial junctions across the shell surface. Consequently, the fabricated substrate exhibits a good SERS performance, achieving an ultra-low limit of detection (LOD) of 10^−11^ M for the standard probe Rhodamine 6G (R6G). Moreover, the macroscopic periodicity of the array ensures large-area signal reproducibility with a relative standard deviation (RSD) of 6.1%. Capitalizing on these robust plasmonic properties, the substrate further demonstrates highly sensitive trace detection of carbendazim with an LOD of 10^−8^ M. By successfully circumventing the conventional trade-off between massive “hot spots” density and scalable uniformity, this work establishes a robust, reliable, and versatile plasmonic design paradigm for advanced environmental pollutant monitoring.

## 2. Materials and Methods

### 2.1. Materials

Carbendazim (≥99.5%), Rhodamine 6G (95%), ethanol (≥99.7%), chloroform (≥99.5%), hydrogen peroxide (35%), acetone (≥99.7%) and concentrated sulfuric acid (95–98%) were all purchased from Sinopharm Chemical Reagent Co., Ltd. (Shanghai, China). Monodisperse polystyrene microbeads (diameter: 500 nm) in an aqueous suspension (2.5 wt%) were purchased from Alfa Aesar (Ward Hill, MA, USA). Quartz substrates were purchased from a commercial supplier in Tianjin, China. Au and Ag targets (purity: 99.99%) and single-crystal silicon wafers were provided by Zhongnuo New Material (Beijing) Technology Co., Ltd. (Beijing, China). All of the chemical reagents were of analytical grade and used without any purification. Deionized water was obtained by a Milli-Q water purification system.

### 2.2. Characterizations

The morphology and structure of the substrates were characterized by scanning electron microscopy (SEM; S-4800, Hitachi, Tokyo, Japan), transmission electron microscopy (TEM; JEM-2010, JEOL Ltd., Tokyo, Japan), and atomic force microscopy (AFM; Icon, Bruker, Billerica, MA, USA). SERS measurements were performed using a confocal micro-Raman spectrometer (XploRA PLUS, HORIBA France SAS, Villeneuve-d’Ascq, France). The optical properties were characterized using a macroscopic angle-resolved spectrometer (R1, Shanghai Ideaoptics Co., Ltd., Shanghai, China). X-ray photoelectron spectroscopy (XPS) measurements were performed using an ESCALAB 250Xi X-ray photoelectron spectrometer (Thermo Fisher Scientific, Waltham, MA, USA). The substrates were prepared using an ion sputtering coater (Q150R Plus, Quorum Technologies Ltd., Laughton, East Sussex, UK) and an oxygen plasma cleaner (CPC-F, CIF International Group Co., Ltd., Beijing, China).

### 2.3. Preparation of Au/Ag Composite Nanoshell Array

**Figure 1:**
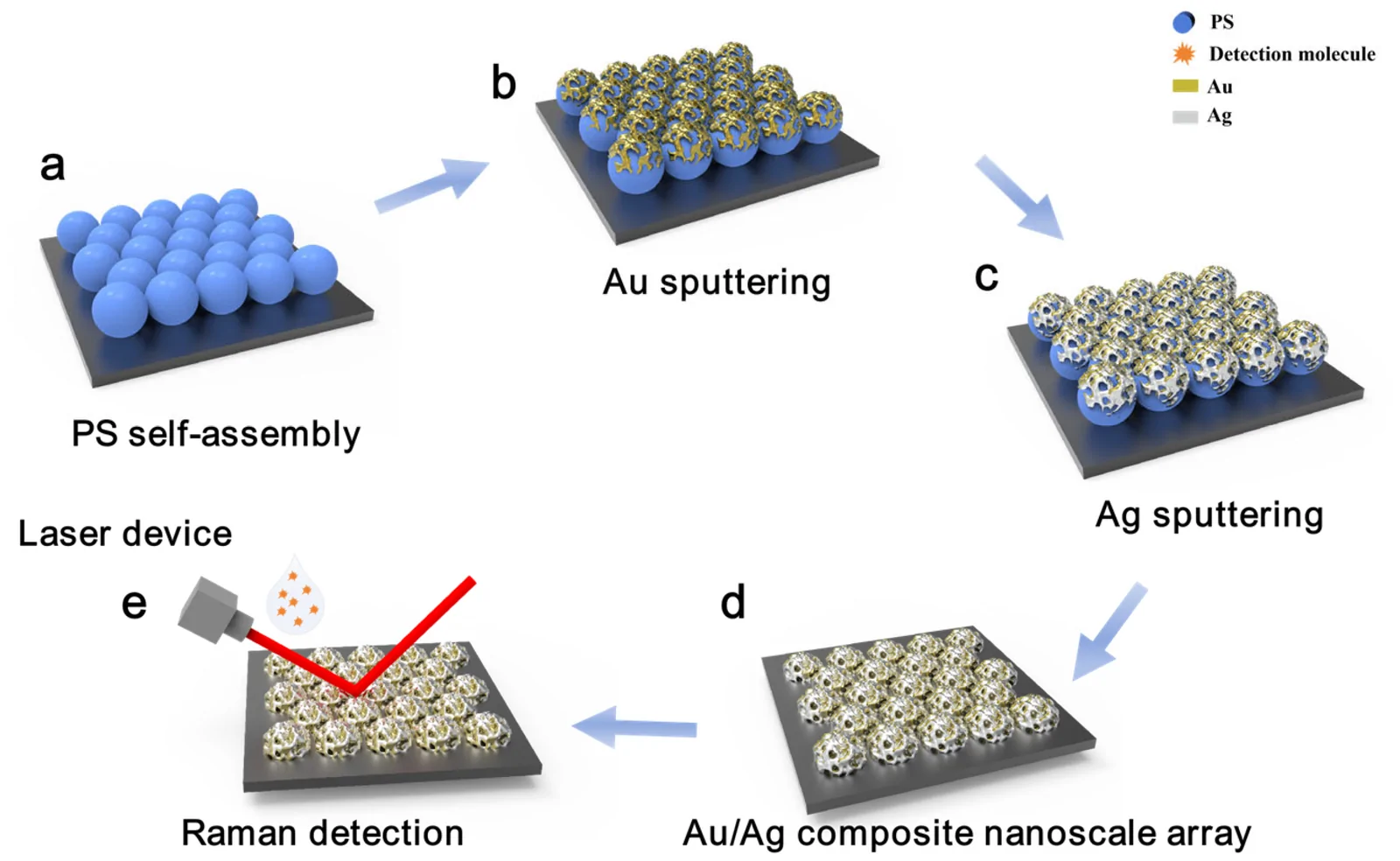
Schematic of the preparation of a Au/Ag composite nanoshell array. (**a**) Self-assembled monolayer of PS microspheres on a silicon wafer. (**b**) Au sputtering on the PS microsphere array. (**c**) Ag sputtering on the Au-modified PS microsphere array. (**d**) PS microsphere template removed through chloroform treatment. (**e**) Au/Ag composite nanoshell array.

Figure 1 illustrates the complete fabrication process for the SERS substrate. Hexagonally close-packed, highly ordered polystyrene (PS) colloidal crystal arrays were fabricated using an air–water interface self-assembly technique. First, the Si wafers and quartz substrates were cleaned in a 1:1 (*v*/*v*) mixture of acetone and ethanol by ultrasonication for 15 min, rinsed thoroughly with deionized water, and dried under a nitrogen stream. The cleaned substrates were subsequently treated with oxygen plasma for 10 min to improve their surface hydrophilicity. The PS microsphere suspension, acetone, and ethanol were then mixed at a volume ratio of 2:1:1 and ultrasonicated to obtain a homogeneous dispersion. The resulting dispersion was carefully introduced onto the water surface, where capillary forces promoted the self-assembly of the PS microspheres at the air–water interface [43,44,45]. After solvent evaporation, an iridescent monolayer of close-packed PS colloidal crystals formed on the water surface and was subsequently transferred onto the Si or quartz substrates using a simple pick-up procedure (Figure 1a and Figure S1). After drying, the metal deposition was conducted via an ion sputtering coater. Metal deposition was performed at a constant sputtering current of 10 mA. Au was sputtered for 5, 10, 15, 20, 25, and 30 s (Figure 1b). Subsequently, Ag was sputtered onto the Au-modified PS microsphere array for 30 s at the same current (Figure 1c). Finally, the PS microsphere template was removed by chloroform (Figure 1d). This straightforward process successfully yielded the final Au/Ag composite nanoshell array. The resulting substrate features a hexagonally close-packed structure, which is highly suitable for subsequent SERS detection (Figure 1e).

### 2.4. SERS Measurement

R6G solutions with varied concentrations (10^−6^–10^−11^ M) were prepared. Before SERS testing, the samples were immersed in the prepared R6G solutions with different concentrations for 30 min to allow the molecules to adsorb onto the Au/Ag composite nanoshell array. During the measurement, an excitation laser at a wavelength of 785 nm was used. The power and integration time are 1 mW and 10 s, respectively. Meanwhile, carbendazim solutions in varied concentrations (10^−5^–10^−8^ M) were prepared. And the sample-treatment and SERS measurement procedures were otherwise kept identical to those used for R6G.

## 3. Results and Discussion

### 3.1. Morphological and Structural Characterization of Au/Ag Composite Nanoshell Array

**Figure 2:**
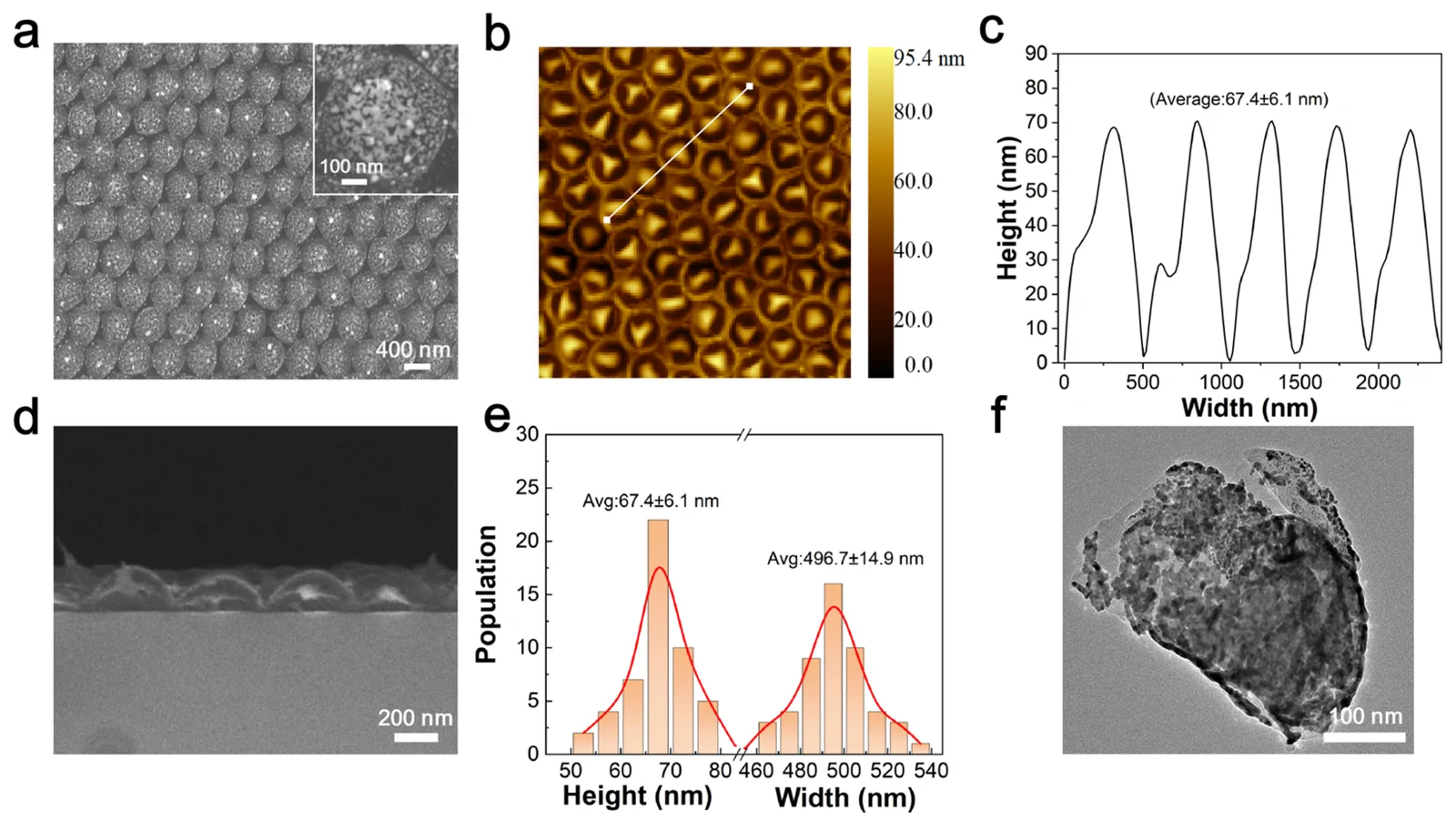
Structural characterization of the Au/Ag composite nanoshell array. (**a**) Low-magnification SEM image of the Au/Ag composite nanoshell array, with an inset showing the surface morphology of a single unit. (**b**) AFM image of the Au/Ag composite nanoshell array. (**c**) AFM height profile along the line marked in (**b**). (**d**) Cross-sectional SEM image of the Au/Ag composite nanoshell array. (**e**) Height and width distributions of 50 randomly selected nanoshell units with corresponding Gaussian fits. (**f**) TEM image of a single Au/Ag composite nanoshell unit.

The morphological and structural features of the Au/Ag composite nanoshell array were systematically characterized. The SEM image reveals that the nanoshells assemble into a hexagonally close-packed array (Figure 2a). The shell surface exhibits pronounced nanoscale roughness, with numerous nanoscale openings, crevices, and junctions between adjacent metallic domains. These morphological features may provide favorable sites for localized electromagnetic enhancement and contribute to the observed SERS response. The AFM image further verifies the ordered and uniform morphology of the Au/Ag composite nanoshell array (Figure 2b). The line-scan profile across five nanoshell units reveals an average height of 67.4 ± 6.1 nm, indicating good dimensional uniformity (Figure 2c). The high-resolution cross-sectional SEM image reveals that the structures remaining after removal of the PS template exhibit shallow dome-like profiles and are attached to the Si substrate (Figure 2d). Rather than retaining the complete 500 nm spherical geometry of the original PS templates, the resulting structures have an average vertical height of 67.4 ± 6.1 nm, consistent with the AFM measurements. This pronounced reduction in height is attributed to the directional sputtering process, which predominantly deposits Au and Ag on the exposed upper portions of the closely packed PS microspheres, thereby producing open-bottom metallic caps. After dissolution of the PS cores in chloroform, the unsupported metallic caps may undergo partial deformation and collapse during solvent evaporation and drying, resulting in the observed shallow dome-like morphology (Figure S2).

Statistical analysis of 50 randomly selected nanostructures revealed an average height of 67.4 ± 6.1 nm and an average lateral width of 496.7 ± 14.9 nm (Figure 2e). Both distributions were well described by Gaussian fits, indicating relatively small dimensional variations across the sample. The TEM image of an individual Au/Ag composite nanoshell unit reveals a hollow cavity surrounded by metallic material (Figure 2f). This observation supports the formation of a hollow nanoshell-like morphology after sequential sputtering and template removal. The bimetallic composition and chemical states of the Au/Ag composite nanoshell array were further investigated by XPS (Figure S3). The Au 4f spectrum exhibits characteristic Au 4f7/2 and Au 4f5/2 peaks at approximately 84.5 and 88.2 eV, respectively, while the Ag 3d spectrum shows Ag 3d5/2 and Ag 3d3/2 peaks at approximately 368.3 and 374.3 eV, respectively. These binding energies are characteristic of predominantly metallic Au and Ag, confirming the coexistence of the two metallic components in the sample.

### 3.2. Morphological Evolution and Optimization of Au/Ag Composite Nanoshell Array

**Figure 3:**
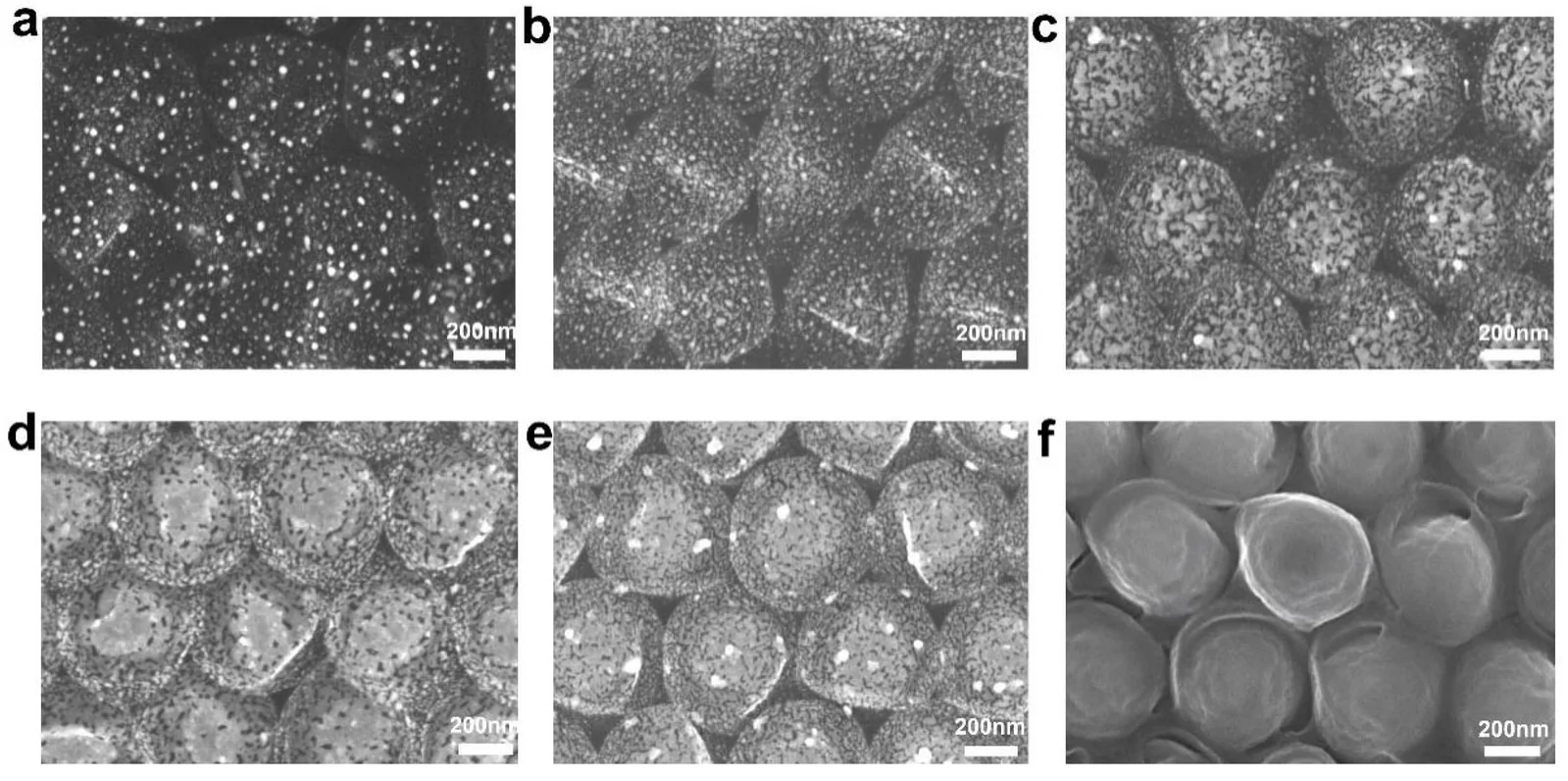
SEM images of Au/Ag composite nanoshell arrays prepared with different Au sputtering times: (**a**–**f**) 5, 10, 15, 20, 25, and 30 s, respectively, with the Ag sputtering time fixed at 30 s.

To optimize the plasmonic response of the Au/Ag nanoshell array, the Au sputtering time was systematically varied from 5 to 30 s in 5 s increments, while the Ag sputtering time was fixed at 30 s (Figure 3). Additional SEM images acquired at a larger field of view are provided in Figure S4. At an Au sputtering time of 5 s, sparsely distributed nanoparticles are observed on the template surface, indicating an early nucleation stage with incomplete surface coverage (Figure 3a). Upon increasing the Au sputtering time to 10 s, a higher particle density and more pronounced surface roughness were observed, consistent with increased metallic coverage and partial coalescence associated with longer Au pre-deposition (Figure 3b).

At an Au sputtering time of 15 s, well-defined nanoshell units with a densely textured surface were formed (Figure 3c). Further increasing the Au sputtering time to 20 and 25 s promoted continued growth and coalescence of the metallic domains (Figure 3d,e). At an Au sputtering time of 30 s, extensive coalescence produces a comparatively smoother metallic structure (Figure 3f). Among the investigated samples, the structure prepared with an Au sputtering time of 15 s and an Ag sputtering time of 30 s exhibited a densely textured surface with abundant nanoscale openings, whereas longer Au sputtering times promoted metallic coalescence and produced a comparatively smoother surface. Combined with the corresponding SERS results, in which the sample prepared with an Au sputtering time of 15 s exhibited the highest Raman intensity, this condition was selected as the optimized configuration under the present experimental conditions. Nevertheless, the current morphological assessment is primarily qualitative, and further quantitative analyses of surface roughness, porosity, and metal coverage would be required to establish a rigorous structure–performance relationship.

### 3.3. SERS Performance Evaluation and Quantitative Detection of R6G

**Figure 4:**
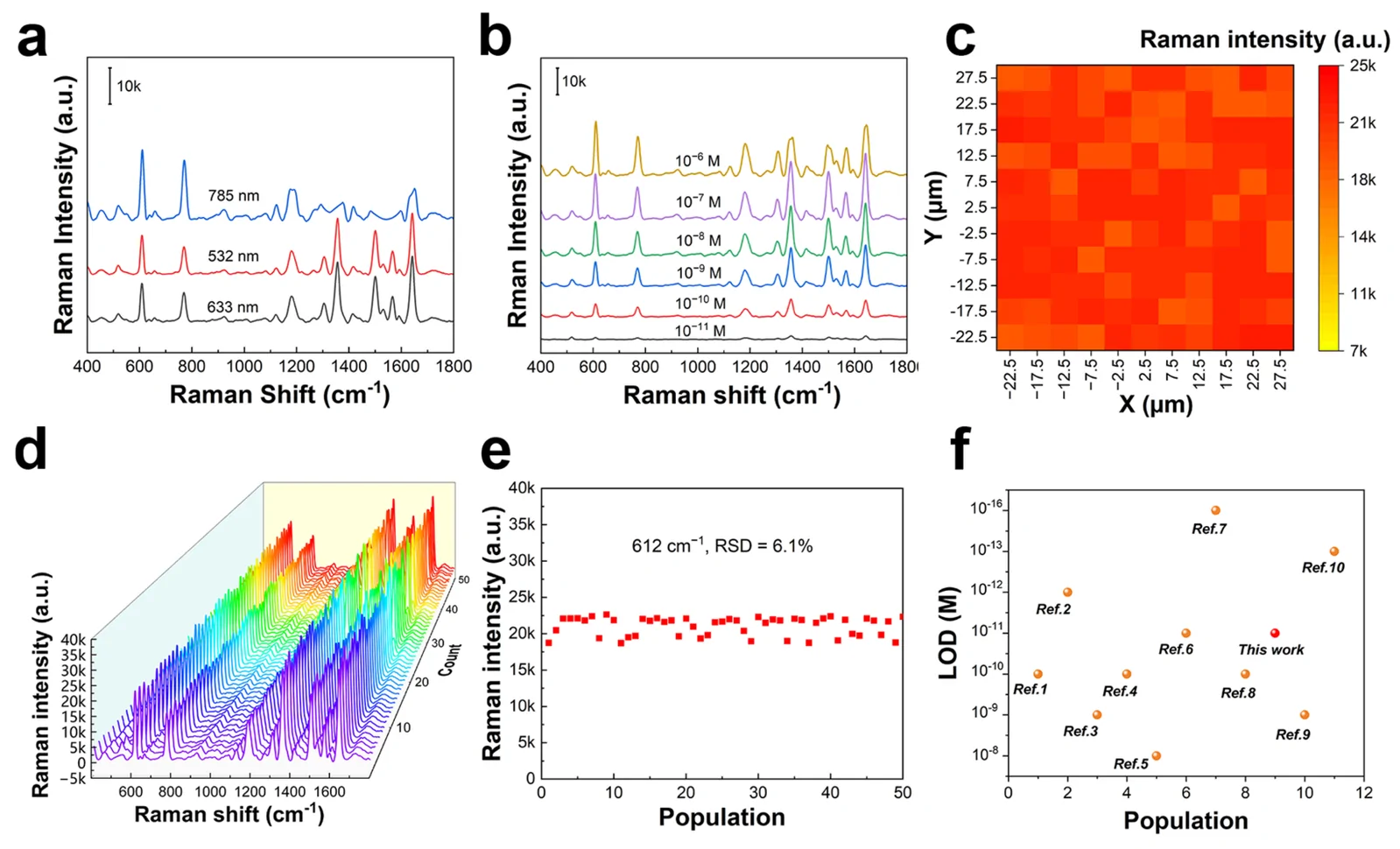
SERS performance of the Au/Ag composite nanoshell array. (**a**) SERS spectra of R6G collected from the Au/Ag composite nanoshell array prepared using Au and Ag sputtering times of 15 and 30 s, respectively, under excitation wavelengths of 532, 633, and 785 nm. (**b**) SERS spectra of R6G at concentrations ranging from 10^−6^ to 10^−11^ M. (**c**) SERS mapping of R6G at 612 cm^−1^ over a 50 × 50 μm^2^ area (step size of 5 μm, integration time of 10 s, and laser power of 1 mW). (**d**) SERS spectra of R6G collected from multiple randomly selected positions for reproducibility evaluation. (**e**) Distribution of Raman intensities at 612 cm^−1^ obtained from 50 measurements, with an RSD of 6.1%. (**f**) Comparison of the R6G detection limit of the present Au/Ag composite nanoshell array with those reported in previous studies.

R6G was employed as the standard probe to evaluate the SERS response of samples prepared with different Au sputtering times under 785 nm excitation (Figure S5). The Raman intensity at 612 cm^−1^ strongly depends on the Au sputtering time and reaches its maximum at 15 s. The SERS signal subsequently decreases with further increases in Au sputtering time. Therefore, the Au/Ag composite nanoshell array prepared with Au and Ag sputtering times of 15 and 30 s, respectively, was selected for all subsequent evaluations. Next, we investigated the effect of the excitation wavelength on this optimized substrate. We measured the SERS spectra of 10^−6^ M R6G on the Au/Ag composite nanoshell array using 532, 633, and 785 nm excitation lasers (Figure 4a). Importantly, the substrate showed clear SERS enhancement under all three wavelengths. These results demonstrate a broad excitation response under the tested experimental conditions. Among the three excitation wavelengths, the highest measured SERS intensity was obtained under 785 nm excitation. To examine the optical response of the substrate, its transmission spectrum was measured over the wavelength range of 400–1000 nm (Figure S6). The Au/Ag composite nanoshell array exhibited a broad optical response extending from the visible to the near-infrared region, with lower transmission at 785 nm than at 532 and 633 nm, suggesting enhanced light–nanostructure interactions in the near-infrared region. Nevertheless, because the measured SERS intensity may also be influenced by wavelength-dependent laser power, optical throughput, detector sensitivity, and the Raman response of R6G, the present results do not establish 785 nm as an intrinsic optimum of the substrate. Therefore, 785 nm was selected for subsequent quantitative measurements only because it produced the highest SERS intensity under the specific experimental conditions used in this study. To further clarify the contributions of the bimetallic composition and nanoshell architecture, control experiments were conducted using Au-only nanoshell arrays, Ag-only nanoshell arrays, and Au/Ag-coated PS arrays before template removal (Figure S7). The Au/Ag composite nanoshell array exhibited the strongest R6G SERS response, indicating that the enhanced performance arises from the combined effects of the Au–Ag bimetallic composition and the hollow nanoshell architecture formed after PS removal.

Figure 4b illustrates the concentration-dependent SERS spectra of R6G solutions ranging from 10^−6^ to 10^−11^ M (mol/L). Although the Raman signal attenuates as the analyte concentration decreases, the characteristic peak at 612 cm^−1^ remains clearly distinguishable even at an ultra-low concentration of 10^−11^ M, demonstrating the advanced trace-detection capability of the platform. Based on the SERS spectrum of 10^−6^ M R6G and the conventional Raman spectrum of 10^−1^ M R6G, an EF of approximately 5.47 × 10^6^ was obtained (Figure S8). Furthermore, plotting the intensity of the 612 cm^−1^ peaks against the logarithm of the R6G concentration yields a strong linear relationship with a correlation coefficient (R^2^) of 0.9970 (Figure S9). This high linearity verifies the substrate’s reliability for precise quantitative analysis. Beyond sensitivity, signal reproducibility and spatial uniformity are critical for practical applications. A large-area SERS mapping measurement over a 50 × 50 μm^2^ region shows a relatively uniform intensity distribution at 612 cm^−1^, with only limited spatial fluctuations, demonstrating good signal uniformity across the substrate (Figure 4c). Furthermore, the reproducibility of the Au/Ag composite nanoshell arrays was evaluated using 50 SERS measurements obtained from five independently fabricated batches prepared on different dates, with 10 randomly selected positions measured for each batch (Figure 4d). The Raman intensity at 612 cm^−1^ was 21,032.8 ± 1282.8 a.u. (mean ± standard deviation), corresponding to an RSD of 6.1%. The 95% confidence interval of the mean intensity was 20,668.2–21,397.3 a.u., and the mean intensities of the five individual batches ranged from 20,724.7 to 21,360.9 a.u. These results demonstrate the good batch-to-batch reproducibility of the Au/Ag composite nanoshell array (Figure 4e).

The short-term storage stability of the Au/Ag composite nanoshell array was further evaluated under ambient laboratory conditions (Figure S10b), with a Ag nanoshell array tested in parallel as a control (Figure S10a). The Raman intensity at 612 cm^−1^ of the Au/Ag composite nanoshell array decreased from 20,757.5 a.u. on day 0 to 20,029.0, 19,026.3, and 18,471.8 a.u. after 3, 6, and 9 days, respectively. Accordingly, approximately 89.0% of the initial SERS intensity was retained after 9 days. In comparison, the intensity of the Ag nanoshell array decreased from 11,921.3 to 9290.4, 8527.3, and 7314.0 a.u., corresponding to a signal retention of only 61.4% on day 9. The substantially smaller intensity loss of the Au/Ag composite nanoshell array indicates improved resistance to ambient aging relative to the Ag-only substrate. These results demonstrate the favorable storage stability of the Au/Ag composite nanoshell array under ambient laboratory conditions.

Finally, the detection performance of the present Au/Ag composite nanoshell array was compared with that of previously reported SERS substrates (Figure 4f). The comparison shows that the present substrate provides a competitive R6G detection limit while simultaneously maintaining good signal reproducibility and a scalable fabrication process, highlighting its potential for practical SERS sensing applications [46,47,48,49,50,51,52,53,54,55].

### 3.4. SERS Detection of Carbendazim and Validation in Apple-Peel Matrix

**Figure 5:**
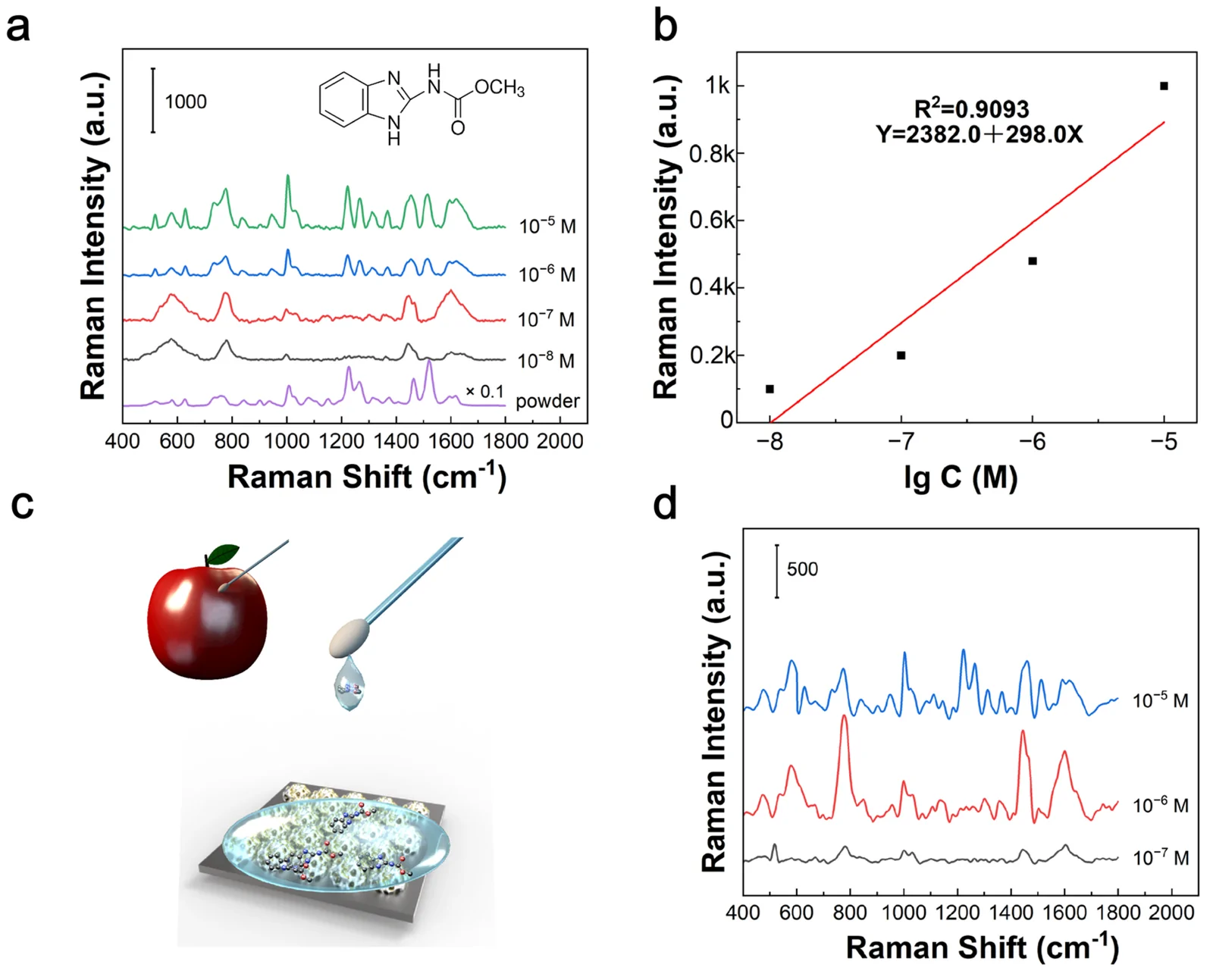
SERS detection of carbendazim using the Au/Ag composite nanoshell array. (**a**) SERS spectra of carbendazim at different concentrations and the conventional Raman spectrum of carbendazim powder. (**b**) Linear relationship between the Raman intensity at 1004 cm^−1^ and the logarithm of carbendazim concentration. (**c**) Schematic illustration of carbendazim detection on apple peel. (**d**) SERS spectra of carbendazim-spiked apple peel samples at different concentrations.

The optimized Au/Ag composite nanoshell array was further applied to the trace detection of carbendazim, a widely used agricultural fungicide. Figure 5a presents the concentration-dependent SERS spectra of carbendazim ranging from 10^−5^ to 10^−8^ M. The characteristic Raman bands gradually decrease in intensity with decreasing carbendazim concentration, while the band at 1004 cm^−1^ remains clearly distinguishable at 10^−8^ M, demonstrating the sensitive detection capability of the substrate.

To further confirm the molecular origin of the observed SERS features, the conventional Raman spectrum of solid carbendazim powder was collected for comparison (Figure 5a). The principal Raman features observed in the SERS spectra are consistent with those of solid carbendazim, particularly the characteristic band at 1004 cm^−1^, supporting the assignment of the detected signals to carbendazim. Quantitative analysis based on the 1004 cm^−1^ band reveals a linear relationship between Raman intensity and the logarithm of carbendazim concentration, with an R^2^ of 0.9093 (Figure 5b).

To further assess the detection performance in a complex food matrix, carbendazim-spiked apple peel samples were analyzed using the Au/Ag composite nanoshell array. The corresponding sampling and SERS detection process is illustrated in Figure 5c. As shown in Figure 5d, the characteristic Raman features of carbendazim remain identifiable in the apple peel matrix over the investigated concentration range. These results demonstrate the feasibility of the Au/Ag composite nanoshell array for trace pesticide detection in an apple-peel matrix and further support its potential for practical food-safety analysis. Future studies may further evaluate the analytical robustness of the platform using additional fruit surfaces, interfering species, and recovery tests under practical sampling conditions.

## 4. Conclusions

In summary, we developed a large-area, highly ordered Au/Ag composite nanoshell array using a scalable gas–liquid interface self-assembly strategy coupled with sequential ion sputtering. Under the optimized sputtering conditions, with Au and Ag deposition times of 15 and 30 s, respectively, the substrate formed a densely textured morphology with abundant nanoscale openings. The resulting nanoscale openings, crevices, and interfacial junctions provide favorable sites for localized electromagnetic enhancement. Consequently, the array exhibited advanced SERS activity, achieving an ultra-low LOD of 10^−11^ M for R6G. The macroscopic periodicity of the array also ensured outstanding spatial uniformity and signal reproducibility, yielding a relative standard deviation RSD of only 6.1%. Furthermore, the substrate enabled sensitive carbendazim detection and successful identification of carbendazim on spiked apple-peel samples. Owing to its simple fabrication, large-area ordered morphology, high SERS sensitivity, and good reproducibility, the Au/Ag composite nanoshell array shows promising potential for rapid screening of pesticide residues on fruit surfaces and related food-safety applications.

## Data Availability

The data that support the findings of this study are available from the corresponding author upon reasonable request.

## Supplementary Materials

The following supporting information can be downloaded at: https://www.mdpi.com/article/10.3390/nano16171109/s1, Figure S1: SEM image of the PS array; Figure S2: Cross-sectional morphology of the Au/Ag composite nanoshell array after PS-template removal; Figure S3: High-resolution XPS spectra of Au 4f and Ag 3d in the Au/Ag composite nanoshell array; Figure S4: SEM images of samples prepared with different Au sputtering times; Figure S5: Effect of Au sputtering time on the SERS performance of the Au/Ag composite nanoshell array; Figure S6: Transmission spectrum of the optimized Au/Ag composite nanoshell array; Figure S7: SERS spectra of R6G obtained using the Au/Ag composite nanoshell array, Au-only nanoshell array, Ag-only nanoshell array, and Au/Ag-coated PS array; Figure S8: Raman spectra used for the calculation of the analytical enhancement factor; Figure S9: Linear relationship between the Raman intensity at 612 cm^−1^ and the logarithm of R6G concentration; Figure S10: Short-term ambient-storage stability of the Au/Ag composite and Ag-only nanoshell arrays.
