# Supplementary material for: Scalable Fabrication of Highly Ordered Au/Ag Composite Nanoshell Array for Ultrasensitive SERS Detection of Trace Pesticides
Source: Nanomaterials (Basel). 2026 Sep 3;16(17):1109. doi: 10.3390/nano16171109 (PMC13567209; doi:10.3390/nano16171109)
Supplement: Supplementary file 1 [file nanomaterials-16-01109-s001.zip › nanomaterials-4467212-supplementary.pdf]

Supplementary Materials

Scalable Fabrication of Highly Ordered Au/Ag Composite Nanoshell Array for Ultrasensitive SERS Detection of Trace Pesticides

Jiabin Chen †, Ye Dong †, Shuyu Jiang, Tao Zhang, Zheng Liu, Kaiwen Hu and Zhiming Chen *

School of Physical Science and Technology, Tiangong University, Tianjin 300387, China
* Correspondence: zmchen@tiangong.edu.cn

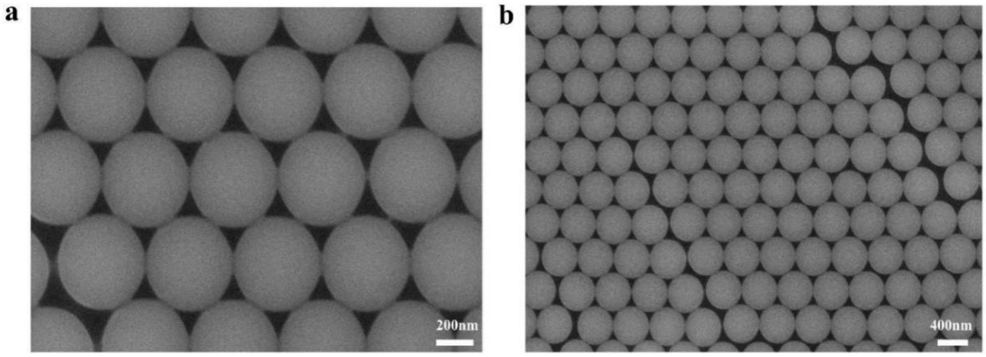

Figure S1. SEM image of PS array.

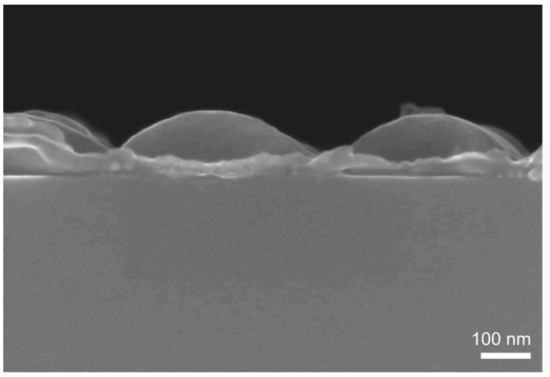

Figure S2. Cross-sectional morphology of the Au/Ag composite nanoshell array after PS-template removal.

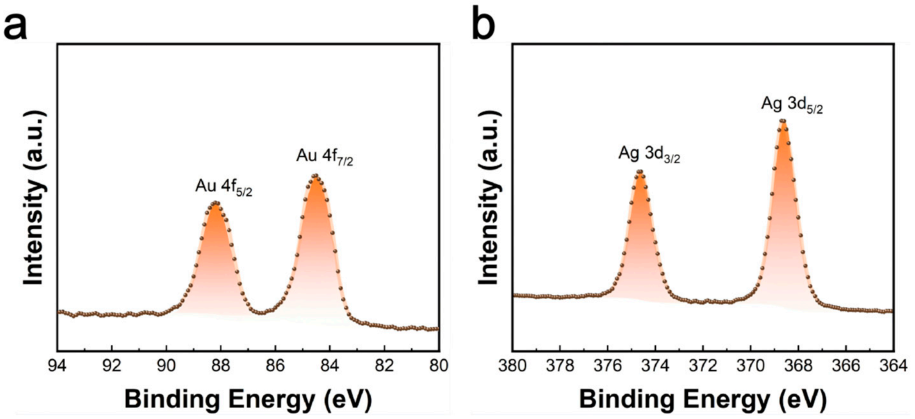

Figure S3. High-resolution XPS spectra of (a) Au 4f and (b) Ag 3d in the Au/Ag composite nanoshell array.

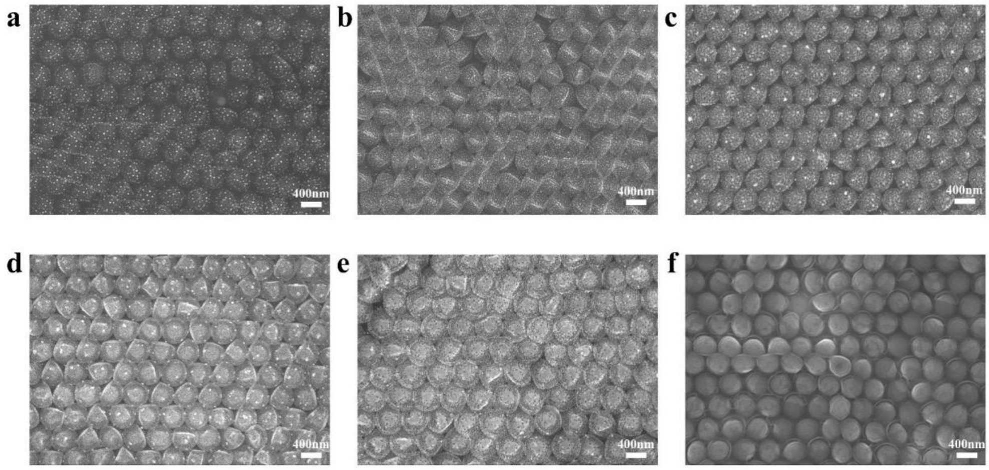

Figure S4. SEM images of the samples deposited with Au sputtering times of (a–f) 5, 10, 15, 20, 25, and 30 s, respectively (20k× magnification).

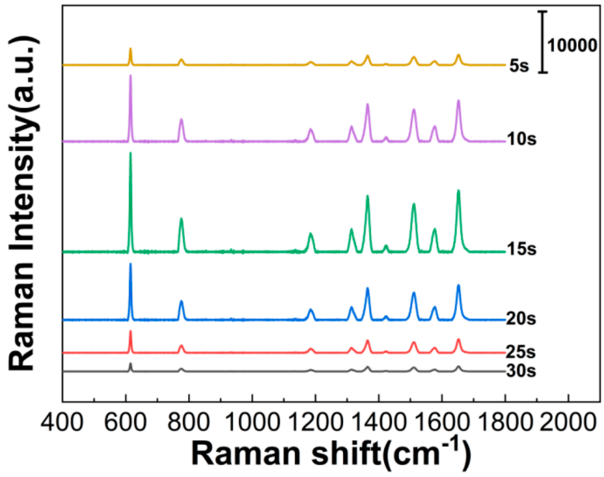

Figure S5. Effect of Au sputtering time on the SERS performance of the Au/Ag composite nanoshell array.

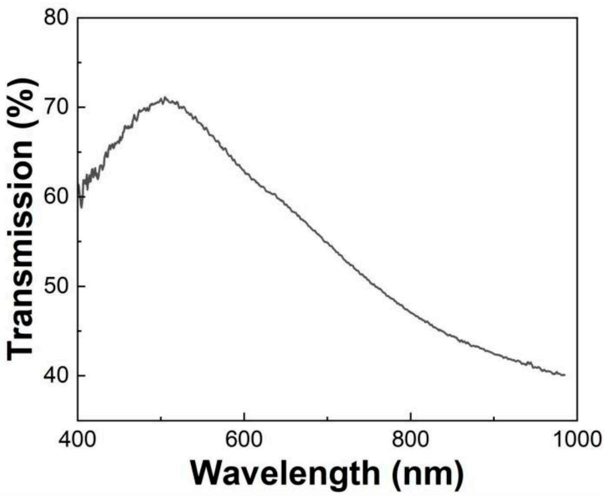

Figure S6. Transmission spectrum of the optimized Au/Ag composite nanoshell array.

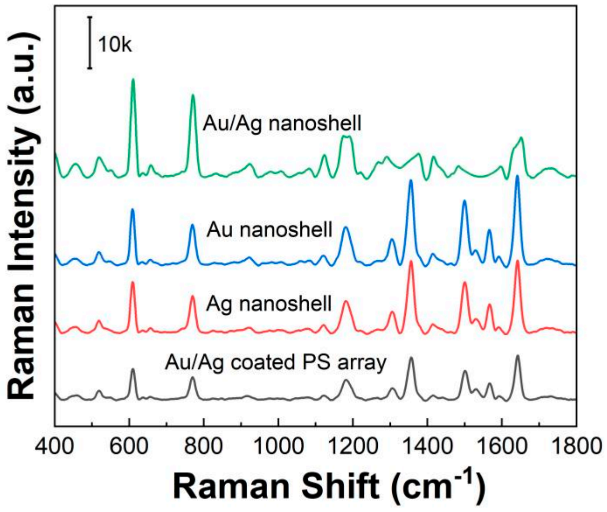

Figure S7. SERS spectra of R6G obtained using the Au/Ag composite nanoshell array, Au-only nanoshell array, Ag-only nanoshell array, and Au/Ag-coated PS array before template removal.

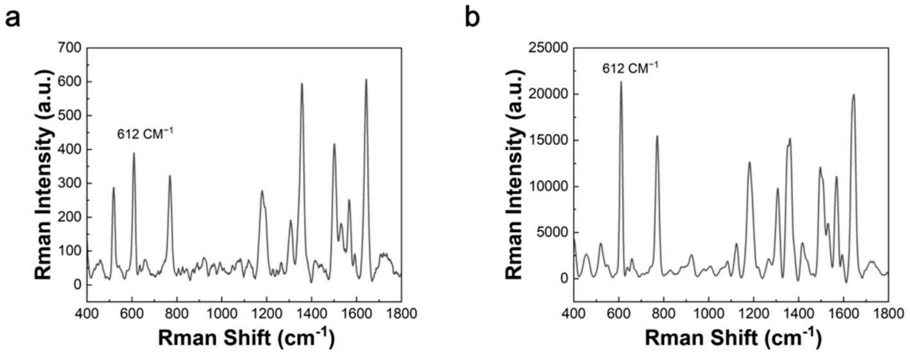

Figure S8. Raman spectra used for the calculation of the enhancement factor (EF): (a) conventional Raman spectrum of 10^{-1} M R6G and (b) SERS spectrum of 10^{-6} M R6G obtained using the Au/Ag composite nanoshell array.

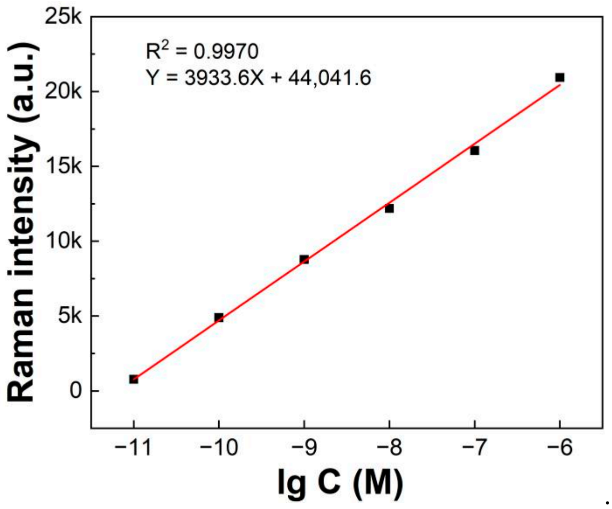

Figure S9. Linear relationship between the peak intensity at 612 cm^{-1} and the logarithm of the R6G concentration in Figure 4b.

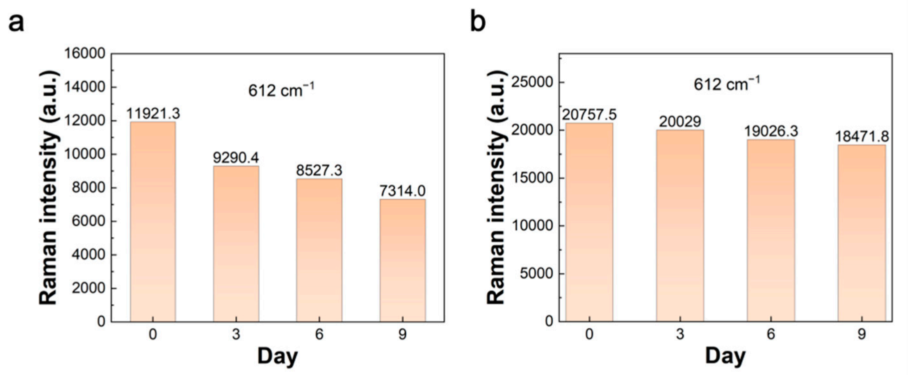

Figure S10. Short-term ambient-storage stability of nanoshell arrays. (a) Raman intensity at 612 cm^{-1} of the Ag-only composite nanoshell array after 0, 3, 6, and 9 days of storage. (b) Raman intensity at 612 cm^{-1} of the Au/Ag nanoshell array after 0, 3, 6, and 9 days of storage.
